# Unusual Manifestation of Dermatomyositis: Exacerbation of Hypertriglyceridemia

**DOI:** 10.7759/cureus.51149

**Published:** 2023-12-27

**Authors:** Hinako Suzuki, Hidesato Odaka, Yuri Takita, Yuka Izumiya, Naoko Shimizu

**Affiliations:** 1 Post Graduate Clinical Education Center, Japanese Red Cross Akita Hospital, Akita, JPN; 2 Department of Respiratory Medicine, Japanese Red Cross Akita Hospital, Akita, JPN; 3 Department of Diabetes, Metabolism and Endocrinology, Japanese Red Cross Akita Hospital, Akita, JPN

**Keywords:** systemic lupus erythematosus, lipoprotein lipase, inflammation, hypertriglyceridemia (htg), dermatomyositis, collagen disease, autoimmune hypertriglyceridemia, anti-lipoprotein lipase antibody

## Abstract

Systemic lupus erythematosus is known to cause autoimmune hyperlipidemia. We present a case in which hypertriglyceridemia was exacerbated by dermatomyositis. A 53-year-old woman with a medical history of undertreated hypertriglyceridemia complained of dyspnea and arthralgia. Despite the treatment, her triglyceride levels increased concurrently with the onset of arthralgia. She had characteristic skin manifestations and tested positive for anti-Jo-1 autoantibodies, leading to a diagnosis of dermatomyositis. Chronic inflammation may result in elevated triglyceride levels. When dermatomyositis is diagnosed, evaluating lipid abnormalities is important.

## Introduction

Dermatomyositis (DM) is an inflammatory autoimmune disorder that affects the skin, muscles, and blood vessels [1]. Moreover, DM is rare, and the prevalence of polymyositis/DM in Japan ranges from 10 to 13 cases per 100,000 population [2]. In contrast, hypertriglyceridemia (HTG) is a common disease. The prevalence of HTG in Japan is 13.6% [3]. HTG in many cases is multifactorial, resulting from the combination of genetic factors and other causes of increased production and/or impaired clearance of triglyceride (TG)-rich lipoproteins [4]. At first glance, no connection appears to be present between the two illnesses. However, 60­­­-70% of patients with DM have increased TG levels [5,6]. Notably, systemic lupus erythematosus (SLE), a collagen disease, can exacerbate HTG [7]. However, whether DM can aggravate HTG remains unknown. In this study, we report the case of a patient with DM, where HTG worsened despite undergoing treatment. In patients with a known autoimmune disease, chronic inflammation may lead to HTG.

## Case presentation

A 53-year-old woman presented with coughing and dyspnea on exertion that had lasted for two weeks. She reported experiencing joint pain in the previous year. Her medical history included HTG for eight years. At initial diagnosis, her TG level was 436 mg/dL. For the first six years, the patient was treated with bezafibrate; however, the drug had to be discontinued owing to the onset of muscle pain. For the next two years, pemafibrate (0.2 mg/day) was administered. Despite undergoing treatment, her condition worsened; her TG level peaked at 1,023 mg/dL one year earlier, concurrently with the onset of arthralgia. No major deterioration in the lifestyle was observed at that time. The patient had a history of smoking 10 cigarettes per day for 33 years (age at 20-53 years of age). The patient had no family history of HTG. At the first visit, the patient's vital signs were as follows: blood pressure, 122/70 mmHg; pulse rate, 100 beats/min; respiratory rate, 18 breaths/min; oxygen saturation, 94% on room air; and temperature, 36.7°C. Although vital signs at rest were relatively good, the patient was hospitalized with post-exertional hypoxemia with an oxygen saturation of 85% on room air. Fine crackles were detected on the dorsal side of the lower lung field. The patient had eyelid edema, a blister-like rash on the flexor aspect of her fingers, and a keratotic rash on the radial side of her index finger. Although the patient experienced arthralgia, she did not experience any spontaneous or gripping muscle pain.

The patient's TG, aspartate aminotransferase, alanine aminotransferase, creatine kinase, and lactate dehydrogenase levels as well as other indicators were abnormal. Especially, the analysis revealed the following: aspartate aminotransferase, 160 IU/L (normal range, 13-30 IU/L); alanine aminotransferase, 130 IU/L (normal range, 7-23 IU/L); creatine kinase, 3820 U/L (normal range, 41-153 U/L); lactate dehydrogenase, 610 IU/L (normal range, 124-222 IU/L); Krebs von den Lungen-6, 983 U/ml (normal range, ≤500 U/L); pulmonary surfactant protein-D, 123 ng/mL (normal range, ≤110.0 ng/mL); TG, 376 mg/dL (normal range, 30-149 mg/dL); and lipoprotein lipase (LPL), 41 ng/mL (normal range, 40-60 ng/mL) (pre-heparin plasma). Urinary protein and occult blood test results were negative. Additional results included C-reactive protein, 0.61 mg/dL (normal range, 0.00-0.14 mg/dL); total cholesterol, 225 mg/dL (normal range, 128-219 mg/dL); high-density lipoprotein (HDL) cholesterol, 30 mg/dL (normal range, 40-96 mg/dL); low-density lipoprotein (LDL) cholesterol, 120 mg/dL (normal range, 60-139 mg/dL); and remnant-like particle cholesterol, 16.6 mg/dL (normal range, 0.0-7.5 mg/dL). The patient was tested positive for antibodies against Jo-1 and aminoacyl transfer RNA synthetase (Table 1).

**Table 1:** Laboratory findings on admission WBC: white blood cell; RBC: red blood cell; HGB: hemoglobin; HCT: hematocrit; PLT: platelet; ESR-1HR: erythrocyte sedimentation rate-1 hour; TP: total protein; Alb: albumin; AST: aspartate aminotransferase; ALT: alanine transaminase; CK: creatine kinase; LDH: lactate dehydrogenase; BUN: blood urea nitrogen; Cr: creatinine; Na: sodium; K: potassium; Cl: chloride; CRP: C-reactive protein; KL-6: Krebs von den Lungen-6; SP-D: surfactant protein D; TSH: thyroid-stimulating hormone; FT4: free thyroxine; FT3: free triiodothyronine; RF: rheumatoid factor; CCP: cyclic citrullinated peptide; ARS: aminoacyl transfer RNA synthetase; Jo-1: histidyl transfer RNA synthetase; MDA5: melanoma differentiation-associated gene 5; TIF1: transcriptional intermediary factor 1; PR3-ANCA: proteinase-3-anti-neutrophil cytoplasmic antibodies; MPO-ANCA: myeloperoxidase-anti-neutrophil cytoplasmic antibodies; HbA1c: hemoglobin A1c; TC: total cholesterol; TG: total triglyceride; HDL-C: high-density lipoprotein cholesterol; LDL: low-density lipoprotein; Apo: apolipoprotein; RLP-C: remnant-like particle cholesterol; LPL: lipoprotein lipase

Laboratory investigation	Results	Reference range
WBC count	7,200/μL	3,300-8,600/μL
Neutrophils	72.4%	41.2-69.7%
Lymphocytes	15.0%	22.1-46.9%
Monocytes	6.7%	4.1-9.6%
Eosinophils	4.8%	0.0-3.5%
Basophils	1.1%	0.0-1.1%
RBC count	484×10^4^/μL	386-492×10^4^/μL
HGB	14.4 g/dL	11.6-14.8 g/dL
HCT	43.0%	35.1-44.4%
PLT	27.2×10^4^/μL	15.8-34.8×10^4^/μL
ESR-1HR	16 mm/hr	≤15 mm/hr
TP	7.1 g/dL	6.6-8.1 g/dL
Alb	3.8 g/dL	4.1-5.1 g/dL
AST	160 IU/L	13-30 IU/L
ALT	130 IU/L	7-23 IU/L
CK	3820 U/L	41-153 U/L
LDH	610 IU/L	124-222 IU/L
BUN	17.0 mg/dL	8.0-20.0 mg/dL
Cr	0.59 mg/dL	0.46-0.79 mg/dL
Na	139 mEq/L	138-145 mEq/L
K	4.3 mEq/L	3.6-4.8 mEq/L
Cl	106 mEq/L	101-108 mEq/L
CRP	0.61 mg/dL	0.00-0.14 mg/dL
KL-6	983 U/L	≤500 U/L
SP-D	123 ng/mL	≤110.0 ng/mL
TSH	1.590 µIU/mL	0.500-5.000 µIU/mL
FT4	1.330 ng/dL	0.900-1.700 ng/dL
FT3	2.960 pg/mL	2.300-4.300 pg/mL
RF	2 IU/mL	0-15 IU/mL
Anti-CCP antibody	0.7 U/mL	≤4.5 U/mL
Anti-nuclear antibody	<40 antibody titer	<40 antibody titer
Anti-ARS antibody	(+)	(-)
Anti-Jo-1 antibody	>550 U/mL	≤10.0 U/mL
Anti-Mi-2 antibody	(-)	(-)
Anti-MDA5 antibody	(-)	(-)
Anti-TIF1-γ antibody	(-)	(-)
PR3-ANCA	<0.5 IU/mL	≤2.0 IU/mL
MPO-ANCA	<0.5 IU/mL	<0.5 IU/mL
Glucose	116 mg/dL	70-109 mg/dL
HbA1c	5.7%	4.6-6.2%
TC	225 mg/dL	128-219 mg/dL
TG	376 mg/dL	30-149 mg/dL
HDL-C	30 mg/dL	40-96 mg/dL
LDL-C	120 mg/dL	60-139 mg/dL
Apo-AⅠ	85 mg/dL	126-165 mg/dL
Apo-AⅡ	17.3 mg/dL	24.6-33.3 mg/dL
Apo-B	114 mg/dL	66-101 mg/dL
Apo-CⅡ	5.1 mg/dL	1.5-3.8 mg/dL
Apo-CⅢ	12.5 mg/dL	5.4-9.0 mg/dL
RLP-C	16.6 mg/dL	0.0-7.5 mg/dL
LPL (pre-heparin plasma)	41 ng/mL	40-60 ng/mL
Urinary protein	(-)	(-)
Urinary occult blood	(-)	(-)

**Figure 1:**
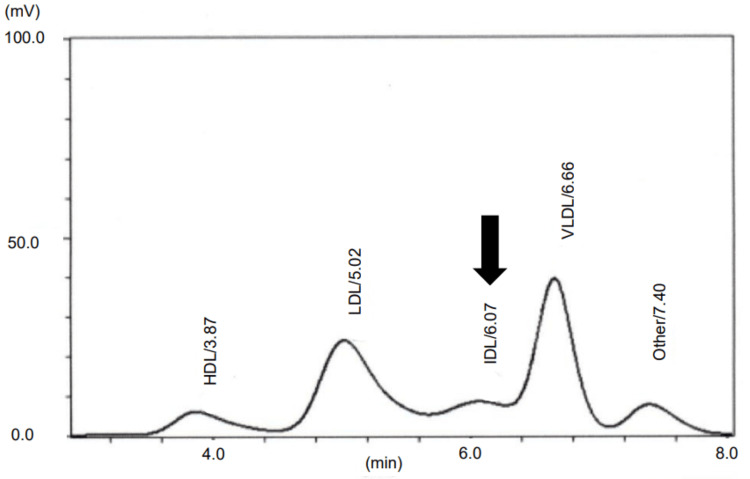
Lipoprotein fraction area ratio determined using high-performance liquid chromatography showing the IDL (arrow) IDL: intermediate-density lipoprotein

The lipoprotein fraction from high-performance liquid chromatography demonstrated elevated levels of intermediate-density lipoprotein (IDL) and very-low-density lipoprotein (VLDL) along with decreased levels of HDL and LDL (Table 2, Figure 1).

**Table 2:** Quantitative value of lipoprotein fraction by high-performance liquid chromatography HDL: high-density lipoprotein; IDL: intermediate-density lipoprotein; LDL: low-density lipoprotein; TC: total cholesterol; VLDL: very-low-density lipoprotein

Laboratory investigation	Results	Reference range
HDL fractionation	8.2%	23.6-49.8%
HDL fixed value	15.0 mg/dL	40.6-91.4 mg/dL
LDL fractionation	34.7%	42.2-63.8%
LDL fixed value	63.8 mg/dL	67.8-132.6 mg/dL
IDL fractionation	11.0%	2.2-6.1%
IDL fixed value	20.2 mg/dL	3.8-12.5 mg/dL
VLDL fractionation	37.7%	2.6-13.9%
VLDL fixed value	69.4 mg/dL	4.9-22.8 mg/dL
Other fractionation	8.5%	0.8-4.4%
Other fixed value	15.6 mg/dL	1.5-9.1 mg/dL
TC fixed value	184 mg/dL	150-219 mg/dL

**Figure 2:**
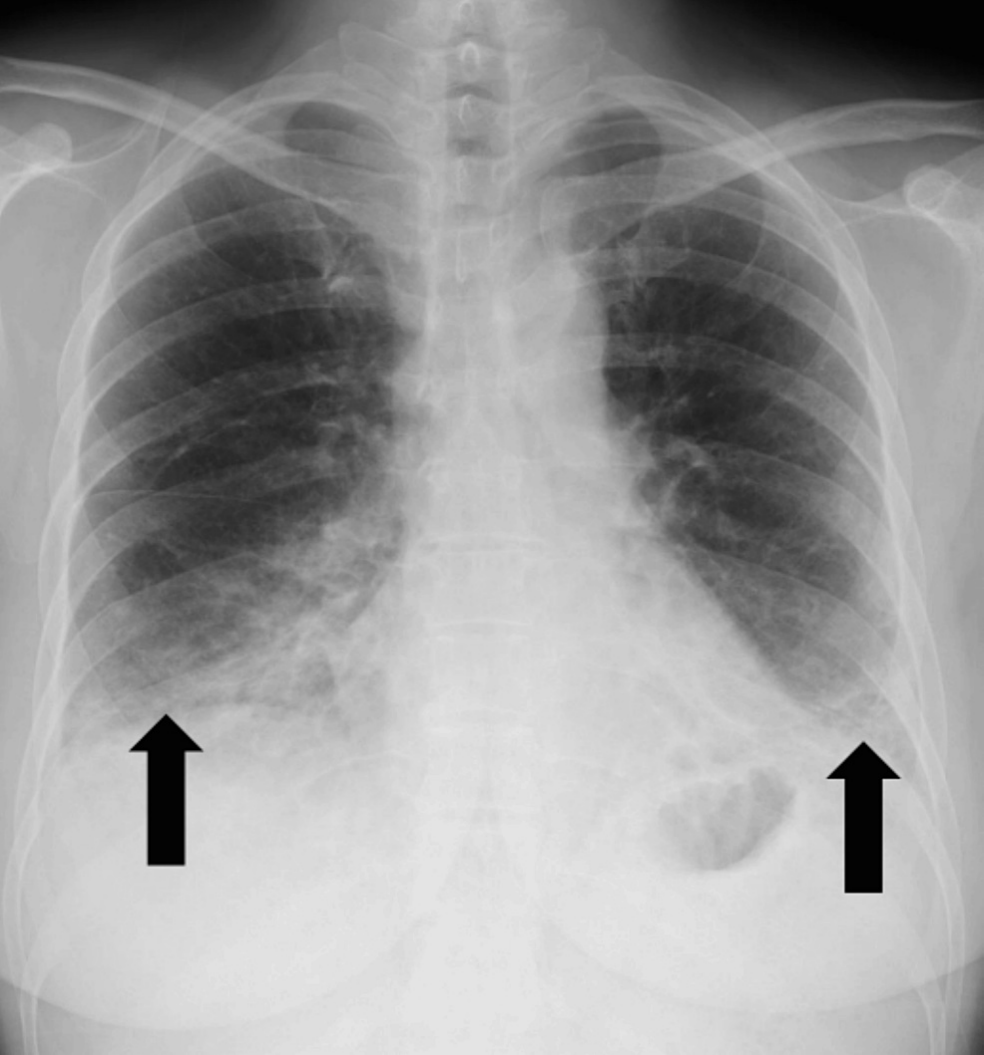
Plain chest radiograph on admission showing interstitial changes (arrow)

Chest radiography revealed interstitial changes (Figure 2).

**Figure 3:**
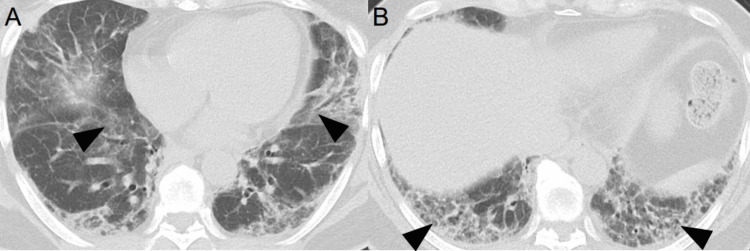
Plain chest computed tomography scan on admission showing A) frosted shadows (arrowhead) and consolidation and B) reticular shadows on the dorsal surfaces of the bilateral lower lung fields (arrowhead)

Chest computed tomography displayed reticular and frosted shadows, traction bronchiectasis, and consolidation of the dorsal surfaces of the lower lungs on both sides (Figure 3A and Figure 3B).

Based on the aforementioned criteria [8], the patient was diagnosed with DM. We hypothesized that her interstitial changes was caused by interstitial pneumonia associated with collagen vascular disease. Additionally, her HTG was considered to have an autoimmune background superimposed on the original underlying HTG.

**Figure 4:**
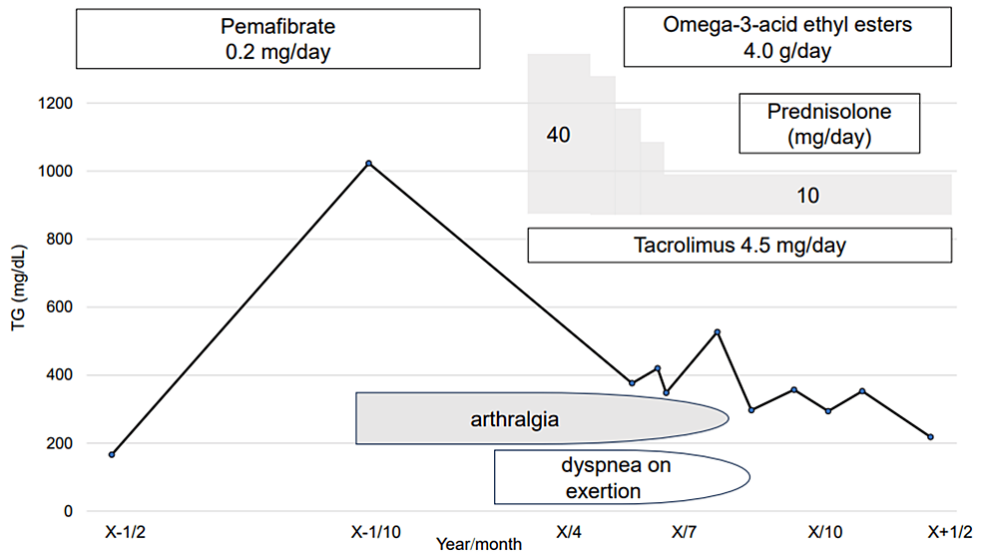
Clinical course. Prednisolone (0.8 mg/kg/day) and tacrolimus (0.075 mg/kg/day) were administered. The patient's symptoms and imaging findings improve; the triglyceride level remains <400 mg/dL

According to the Japanese guidelines [9,10], treatment involved prednisolone (0.8 mg/kg/day) and tacrolimus (0.075 mg/kg/day). The patient's symptoms and imaging findings were improved, and the TG levels remained below 400 mg/dL (Figure 4).

## Discussion

Here, we learned about two significant clinical issues. First, increased TG levels may be an indicator of worsening inflammation. Second, when DM is diagnosed, evaluating the serum lipid profiles is important.

Elevated TG Levels May Be an Indicator of Worsening Inflammation

HTG occurs because of dysfunctional LPL-mediated lipolysis [11]. Patients with SLE demonstrate a marked decrease in LPL activity; approximately 40% have anti-LPL antibodies [12], which positively correlate with TG levels. The "lupus pattern" of dyslipoproteinemia is characterized by elevated VLDL and TG levels and reduced HDL levels [7,13]. Active SLE can increase the VLDL and TG levels and decrease the HDL and LDL levels compared to inactive SLE [7].

Dyslipoproteinemia is a common feature in patients with DM that is characterized by an increase in TG and a decrease in HDL levels [5]. Elevated TGs and low HDL levels were also observed in our case. Moreover, in our case, the IDL levels were elevated. Causes of secondary dyslipidemia with increased IDL levels include autoimmune disease, multiple myeloma, monoclonal gamma globulinemia, and hypothyroidism [14]. The time of onset of arthralgia, a DM symptom, coincided with the time of TG exacerbation in our case. According to Kozu et al. and Eimer et al., dyslipidemia in DM was associated with disease activity [15,16]. In our case, an exacerbation of HTG may have been caused by increased DM activity. Inflammation might partly account for the changes in serum lipid profiles in DM [5].

Concerning anti-LPL antibodies, measuring the anti-LPL antibody levels is not possible in daily clinical practice, including our case. Opinions are divided regarding its existence. In the work by Reichlin et al., sera samples were obtained from patients with polymyositis and DM (n=30); interestingly, 12 (40%) were positive for reactivity with LPL [17]. In contrast, Cotrim et al. did not observe the presence of the anti-LPL antibody in DM, and their results demonstrated that anti-LPL antibodies are not implicated in the pathophysiology of dyslipidemia in patients with DM [18]. Moreover, they mentioned that the alternations identified in the TG and HDL levels may be associated with the decrease in the activity of LPL [19]. However, measuring the LPL activity levels is not possible in daily clinical practice. Further investigation is required since reports on autoimmune HTG due to DM are limited.

When DM Is Diagnosed, Evaluating the Serum Lipid Profiles Is Essential

Dyslipoproteinemia is a common feature in patients with DM that is characterized by an increase in TG and a decrease in HDL levels [5], suggesting a high risk of atherosclerosis and cardiovascular diseases. Although in our case the patient originally had HTG, we re-evaluated her condition. The present case also met these characteristics. Metabolic syndrome, including dyslipidemia, is highly prevalent in DM cases (41.7%) [20]. Therefore, when DM is diagnosed, we should consider the evaluation of the serum lipid profiles.

## Conclusions

Elevated TG levels may indicate worsening inflammation, and when DM is diagnosed, evaluating the serum lipid profiles is important. Especially, it might be beneficial to assess dyslipidemia when inflammatory autoimmune disorders, including DM, worsen. Some cases of elevated TG owing to worsening inflammation may remain unrecognized, suggesting that a considerable number of hidden cases may exist with rising TG levels. Accumulating further reports is essential to ascertain whether hidden cases of rising TG are more commonly encountered. This is crucial for elucidating the mechanisms of HTG in inflammatory autoimmune disorders.
